# Conventional and contrast-enhanced ultrasound in the differential diagnosis of recurrent dermatofibrosarcoma protuberans and postoperative scar

**DOI:** 10.1186/s12885-024-11991-7

**Published:** 2024-03-04

**Authors:** Xia Gong, Jia Li, Angang Ding, Jiaxin Zuo, Yamin Rao, Jun Chen, Ping Xiong

**Affiliations:** 1 Department of Ultrasound, Shanghai Ninth People’s Hospital, School of Medicine, Shanghai Jiaotong University, Shanghai, P. R. China; 2 Department of Pathology, Shanghai Ninth People’s Hospital, School of Medicine, Shanghai Jiaotong University, Shanghai, P. R. China; 3 Department of Dermatology and Dermatologic Surgery, Shanghai Ninth People’s Hospital, School of Medicine, Shanghai Jiaotong University, Shanghai, P. R. China

**Keywords:** Contrast-enhanced ultrasound, Conventional ultrasound, Color Doppler, Postoperative scar, Recurrent dermatofibrosarcoma protuberans

## Abstract

**Background:**

Dermatofibrosarcoma protuberans (DFSP) has a high recurrence rate after resection. Because of the lack of specific manifestations, recurrent DFSP is easily misdiagnosed as post-resection scar. A few series have reported ultrasound findings of recurrent DFSP; moreover, the usefulness of contrast-enhanced ultrasound in differentiating recurrent DFSP has not been studied.

**Objective:**

We investigated conventional and contrast-enhanced ultrasound in the differential diagnosis of recurrent DFSP and post-resection scar.

**Methods:**

We retrospectively evaluated the findings of conventional and contrast-enhanced ultrasound in 34 cases of recurrent DFSP and 38 postoperative scars examined between January 2018 and December 2022.

**Results:**

The depth and vascular density of recurrent DFSP were greater than those of postoperative scars (*P* < 0.05). On gray-scale ultrasound, recurrent DFSP lesions were more commonly irregular, heterogeneous, and hypoechoic, with finger-like projections and ill-defined borders. Postoperative scar was more likely to appear as hypoechoic and homogeneous with well-defined borders (*P* < 0.05). On color Doppler ultrasound, recurrent DFSP was more likely to feature rich arterial and venous blood flow, and postoperative scar was more likely to display poor blood flow (*P* < 0.05). On contrast-enhanced ultrasound, recurrent DFSP was more likely to feature heterogeneous hyper-enhancement, and postoperative scar was more likely to display homogeneous iso-enhancement (*P* < 0.05). Recurrent DFSP presented a higher peak and sharpness than postoperative scar (*P* < 0.05).

**Conclusion:**

Conventional and contrast-enhanced ultrasound produced distinct features of recurrent DFSP and post-resection scar, which could improve the accuracy of differential diagnosis.

## Introduction

Dermatofibrosarcoma protuberans (DFSP) is a rare, low- to intermediate-grade sarcoma that appears as an asymptomatic, red-pink, indurated plaque growing into multiple nodules over a period of time [1]. In the early stages of DFSP, lesions are small in size and limited to the dermal layer. With progression, the tumor tends to invade deep tissue, muscle, and even bone, complicating the complete removal of the tumor and leading to a high recurrence rate after surgery [2, 3].

DFSP has nonspecific characteristics and can easily be mistaken for other superficial masses such as epidermal cysts, lipoma, and dermatofibroma. In a retrospective study involving 214 cases [4], more than half of patients with DFSP experienced one or more misdiagnoses, which may lead to recurrence after local excision. Because of the lack of specific characteristics, recurrent DFSP is easily misdiagnosed as post-resection scar in the early stage and must be definitively diagnosed according to imaging and pathology.

Ultrasound (US) examination is a rapid, accessible, and inexpensive first-line modality for evaluating cutaneous and subcutaneous mass lesions. It provides valuable information regarding diagnosis of DFSP, assessment of lesion extent, and monitoring of response to therapy. Contrast-enhanced ultrasound (CEUS) is widely used in clinical diagnosis of abdominal and superficial organ tumors and in differentiation of benign from malignant tumors [5, 6]. There have been several reports on the US features of DFSP [7–13]. In addition, two reports [14, 15] found that CEUS provided a new method for locating and predicting the size of DFSP tumors, and CEUS displayed higher concordance than US with histology regarding maximum diameter and depth [15].

In this study, we present the conventional US and CEUS findings for recurrent DFSP and post-resection scar, and the utility of each modality in differential diagnosis, with the aim of better determining DFSP recurrence.

## Materials and methods

### Patients

The authors’ institutional review board approved the retrospective collection and analysis of data, and the study protocol was approved by the ethics committee (2017-451-T3347). The requirement for patient informed consent was waived due to the study’s retrospective design.

We retrieved and analyzed the preoperative ultrasonographic data for 34 recurrent DFSPs (patients with a negative pathological margin at the last surgery) and 38 post-resection scars (patients with DFSP post-resection at our institution) evaluated between January 2018 and December 2022. Diagnoses of DFSP were pathologically confirmed, and postoperative scars were confirmed by follow-up of more than 1 year. Items reviewed in the medical records of each patient included age, sex, clinical presentation, onset, and lesion location. The patient follow-up intervals ranged from 6 months to 2 years (mean, 1 year).

### US examination

Ultrasonography was performed before treatment using the GE Voluson E8 (GE Healthcare, Austria) and MyLab Class C (Esaote, Genoa, Italy), with a broadband (9–14 MHz) linear transducer. The lesions were evaluated using conventional US (gray-scale, color Doppler) and CEUS. Imaging assessment of all patients was performed by two US specialists with 5 years of experience who were blinded to histopathological findings; a consensus was then reached.

On gray-scale US images, the lesion size, depth (when extended field-of-view US was inadequate to measure lesion size), echotexture (homogeneous or heterogeneous), echogenicity compared with adjacent muscle (hyperechoic, isoechoic, hypoechoic, mixed hyper- and hypoechoic), and margins (well-defined or ill-defined) were evaluated. In color Doppler US, the B-mode display was overlaid with additional color pixels to assess the presence and features of blood flow at a given time. Color velocity imaging was performed using a constant velocity scale (± 6 cm/s) [16]. Vessel density was estimated by counting the number of vessels per square centimeter outlined on color Doppler flow imaging (CDFI) [17]. The CDFI diagnostic criteria are as follows: Adler 0, no vascularization; Adler 1, vascularization not rich; and Adler 2 or 3, rich vascularization [18]. Arteriovenous spectrum and blood flow velocity (arterial and venous) were determined using pulse Doppler US.

CEUS was performed using the Esaote MyLab™ Twice ultrasound system (Esaote) equipped with a 7–12 MHz high-energy linear probe. The lesion in question was first examined using conventional US and then with CEUS. A suspension of the contrast agent was obtained by adding 5 mL of physiological saline to SonoVue (Bracco SpA, Milan, Italy). A contrast bolus of 3 mL was injected into the median cubital vein, followed by a 5 mL saline flush. The DICOM dynamic data were then stored. Each contrasted imaging acquisition lasted for at least two continuous minutes, and processing was performed using QontraXt software (Esaote).

On CEUS images, the DFSPs were evaluated for the following characteristics: homogeneity of enhancement, classified as homogeneous or heterogeneous; enhancement intensity (using soft tissue around the lesion as a reference), classified as iso-enhancement, hyper-enhancement, hypo-enhancement, or no enhancement; peak time of contrast enhancement; peak; and sharpness (a small slope of the ascending branch indicates a flat curve, a large slope of the ascending branch indicates a steep curve).

### Data analysis

Statistical evaluation was performed using SPSS version 23.0 (IBM Corporation, Armonk, NY, USA). One-way analysis of variance (ANOVA), the Student’s t-test, and the χ^2^ test were used to analyze the US findings for recurrent DFSP and postoperative scars; statistical significance was set at a P value < 0.05.

## Results

### Patient characteristics

Clinical data such as age, sex, lesion type, and lesion location are summarized in Table 1. All 34 recurrent DFSP lesions were treated using Mohs micrographic surgery.

**Table 1:** Clinical characteristics of recurrent dermatofibrosarcoma protuberans (DFSP) and post-resection scar

Variables	Recurrent DFSP	Post-resection scar	*P*
Case No.	34	38	
Sex			0.170
Male	18 (52.9)	14 (32.8)	
Female	16 (47.1)	24 (63.2)	
Type of lesion			
Plaque	17 (50)	27 (71.1)	0.067
Red nodule	5 (14.7)	0	0.047
Red protrusion	12 (35.3)	11 (28.9)	0.564
Age, years			0.647
Mean	35.8 ± 12.8	31.0 ± 14.4	
Range	7–59	4–55	
Region			
Head and neck	4 (11.8)	2 (5.3)	0.569
Trunk	26 (76.4)	30 (78.9)	0.801
Extremities	4 (11.8)	6 (15.8)	0.879

Categorical variables are presented as number (%)

### US findings

**Fig. 1:**
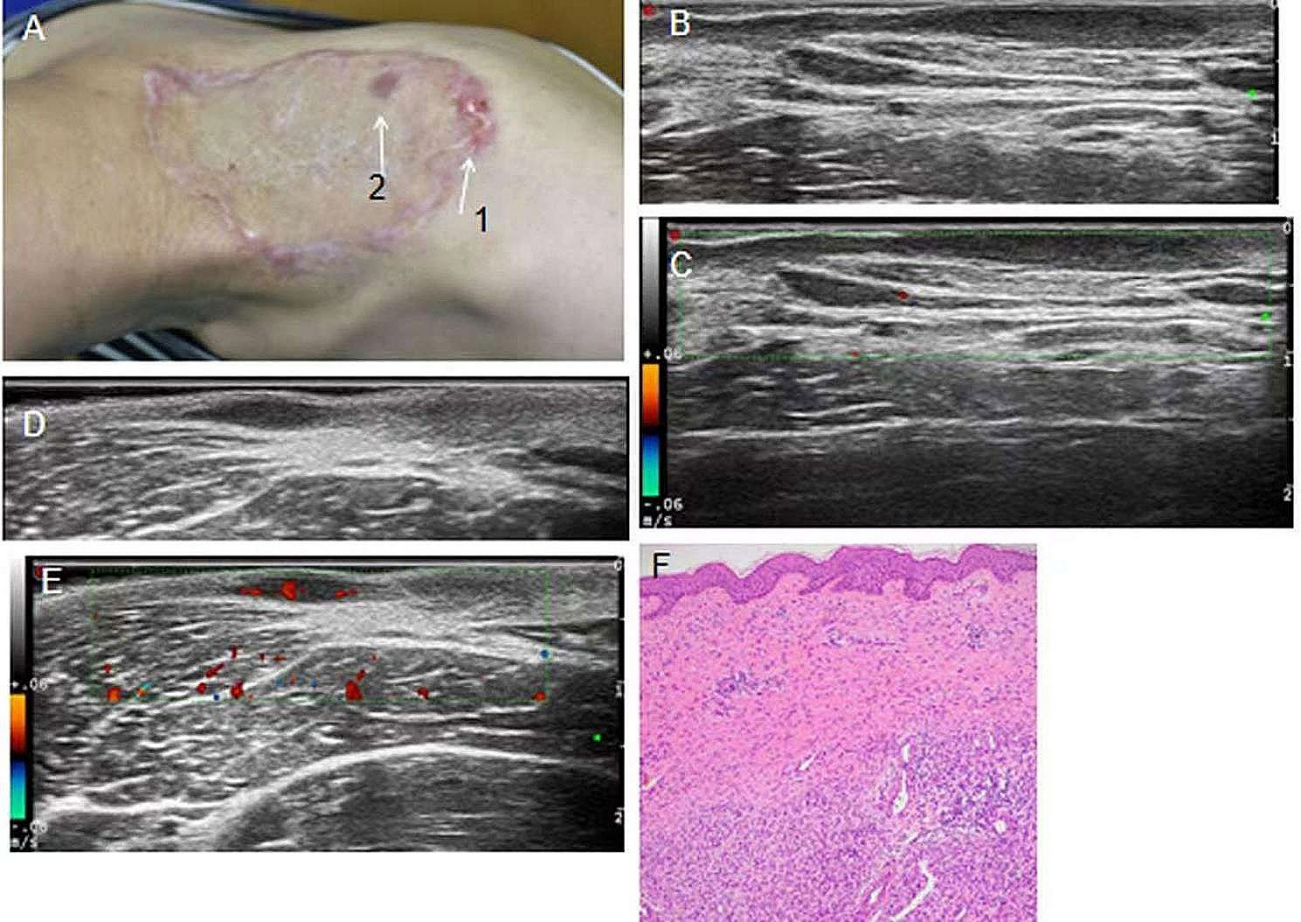
**A** Recurrent DFSP and post-resection scar with two protrusions in the left shoulder of a 28-year-old woman. **B** Transverse ultrasonogram (No. 1) revealing a well-defined, hypoechoic, homogeneous, and subcutaneous lesion. **C** Color Doppler ultrasonogram revealing no blood flow signal. **D** Transverse ultrasonogram (No. 2) revealing an well-defined, hypoechoic, homogeneous, and subcutaneous lesion. **E** Color Doppler ultrasonogram revealing rich blood flow signal with vascular densities reaching 3/cm^2^. **F** Histology showing a storiform growth pattern and proliferation of vessels (hematoxylin-eosin ×10)

**Fig. 2:**
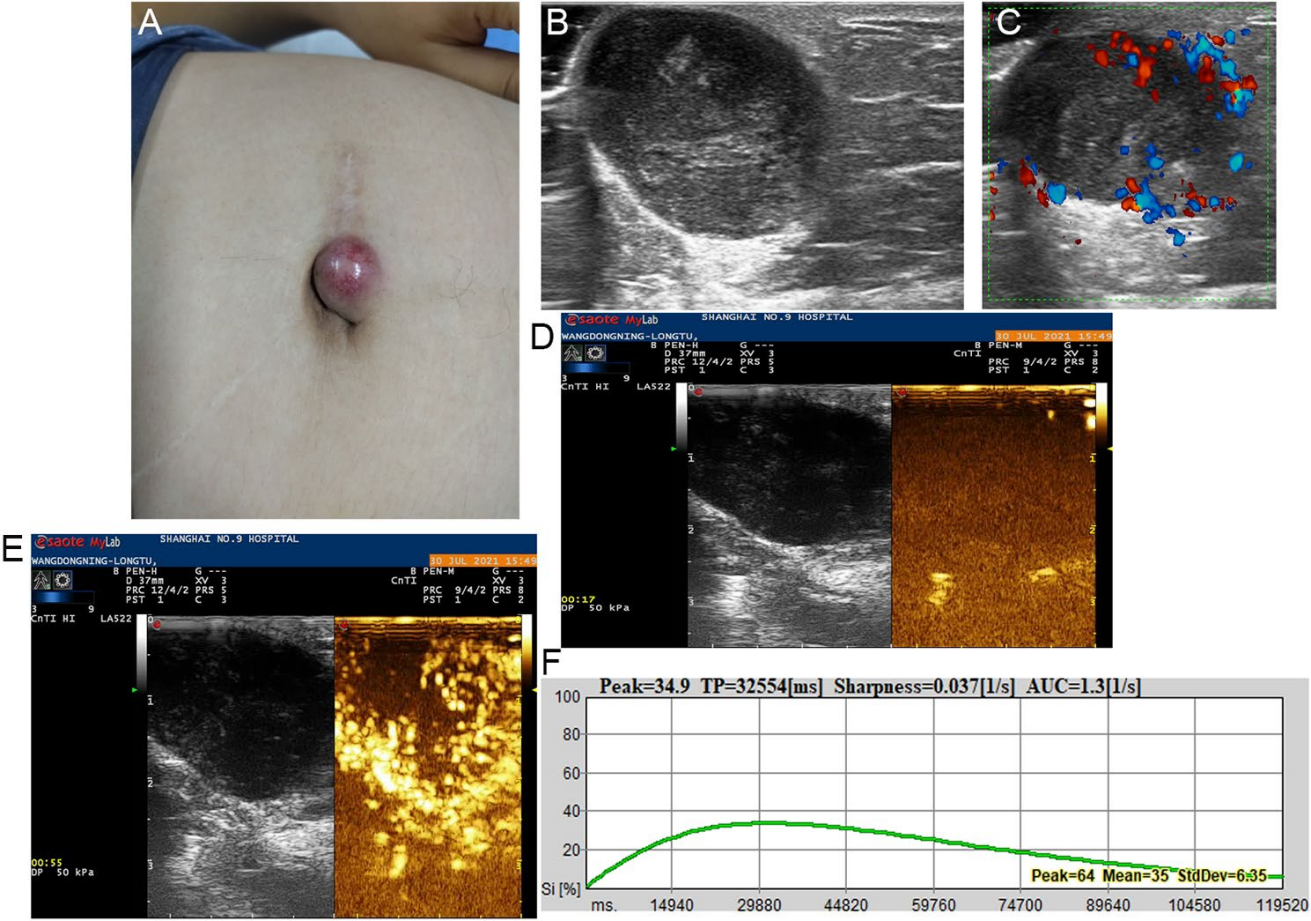
**A** Recurrent DFSP with red nodule in the abdominal wall of a 36-year-old man. **B** Transverse ultrasonogram revealing a well-defined, hypoechoic, heterogeneous, and subcutaneous lesion. **C** Color Doppler ultrasonogram revealing rich vascularization with vascular densities reaching 4.5/cm^2^. **D** Contrast-enhanced ultrasound revealed the trend that contrast agent enters the lesion from the periphery to the center. **E** Contrast-enhanced ultrasound revealed heterogeneous hyper-enhancement at peak (the 32th second), with filling defect in the center. **F** Time intensity curve revealed peak of 34.9 and sharpness of 0.037

**Fig. 3:**
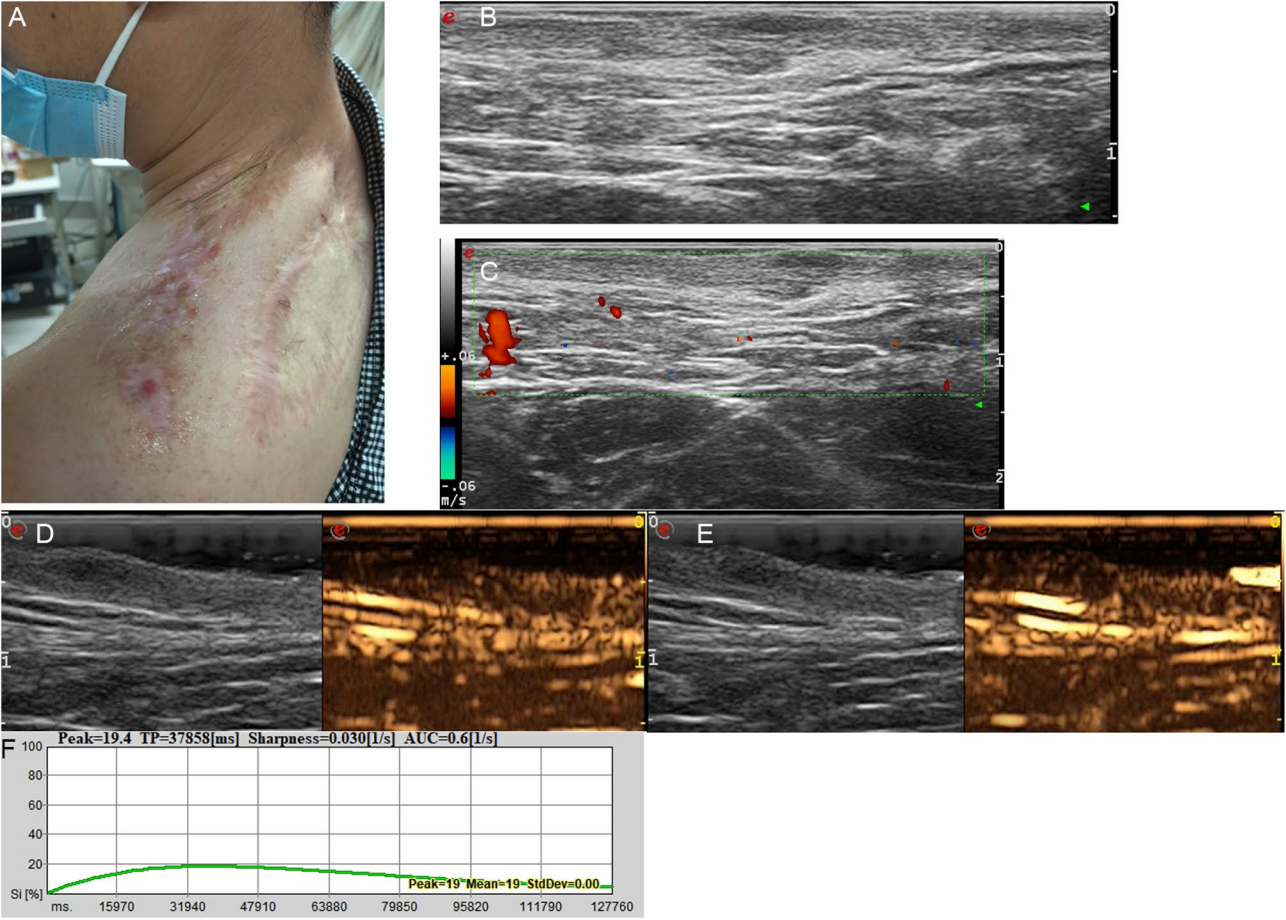
**A** Post-resection scar with red protrusion in the left shoulder of a 46-year-old man. **B** Transverse ultrasonogram revealing a well-defined, hypoechoic, homogeneous, and subcutaneous lesion. **C** Color Doppler ultrasonogram revealing no blood flow signal. **D** Contrast-enhanced ultrasound revealed the trend that contrast agent enters the lesion from the bottom. **E** Contrast-enhanced ultrasound revealed homogeneous hyper-enhancement at peak (the 38th second). **F** Time intensity curve revealed peak of 19.4 and sharpness of 0.030

**Fig. 4:**
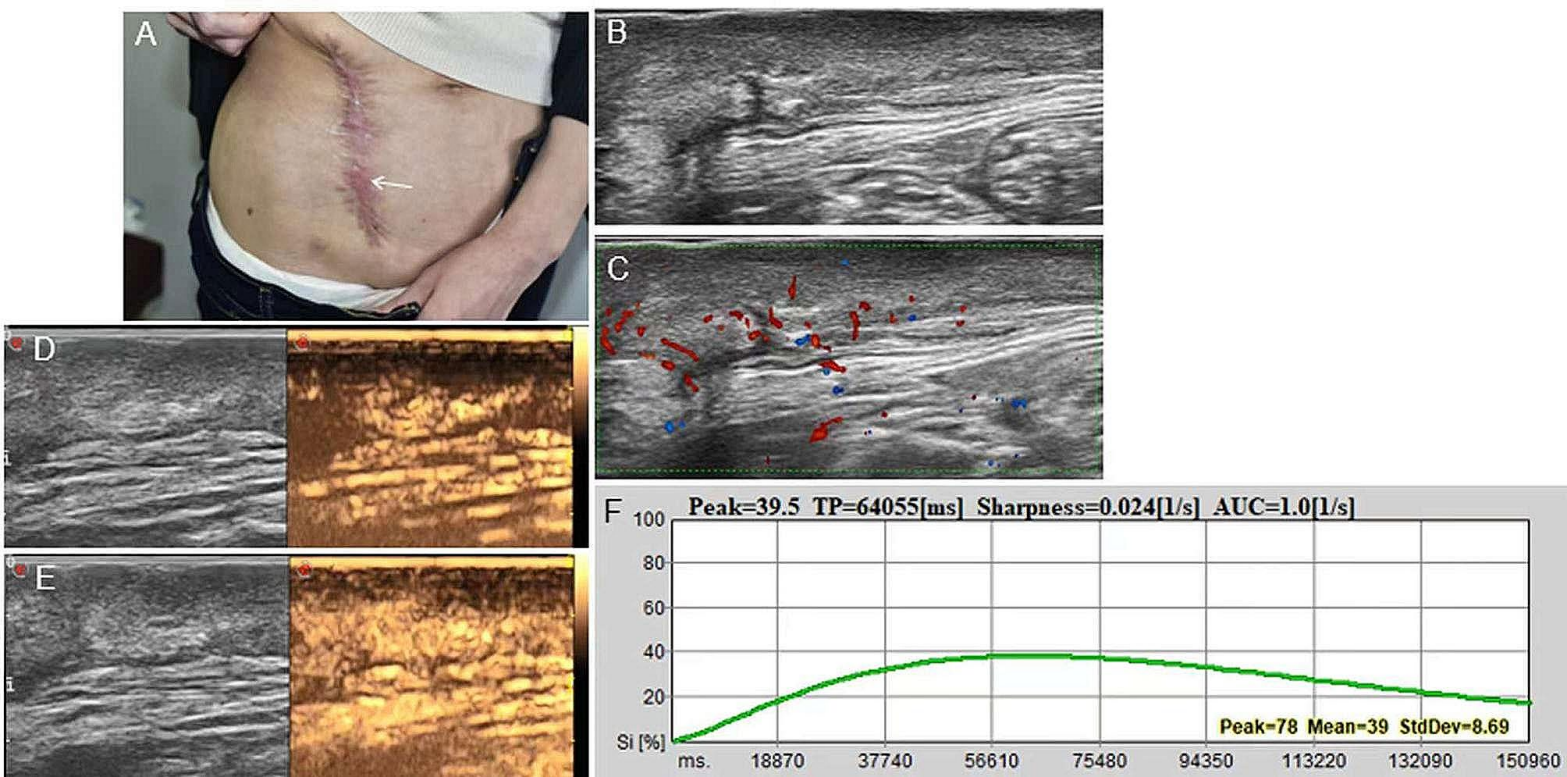
**A** Post-resection scar with red protrusion in the abdominal wall of a 41-year-old woman. **B** Transverse ultrasonogram revealing a well-defined, hypoechoic, homogeneous, and subcutaneous lesion. **C** Color Doppler ultrasonogram revealing rich blood flow signal. **D** Contrast-enhanced ultrasound revealed the trend that contrast agent enters the lesion from the bottom. **E** Contrast-enhanced ultrasound revealed homogeneous hyper-enhancement at peak (the 64th second). **F** Time intensity curve revealed peak of 39.5 and sharpness of 0.024

**Fig. 5:**
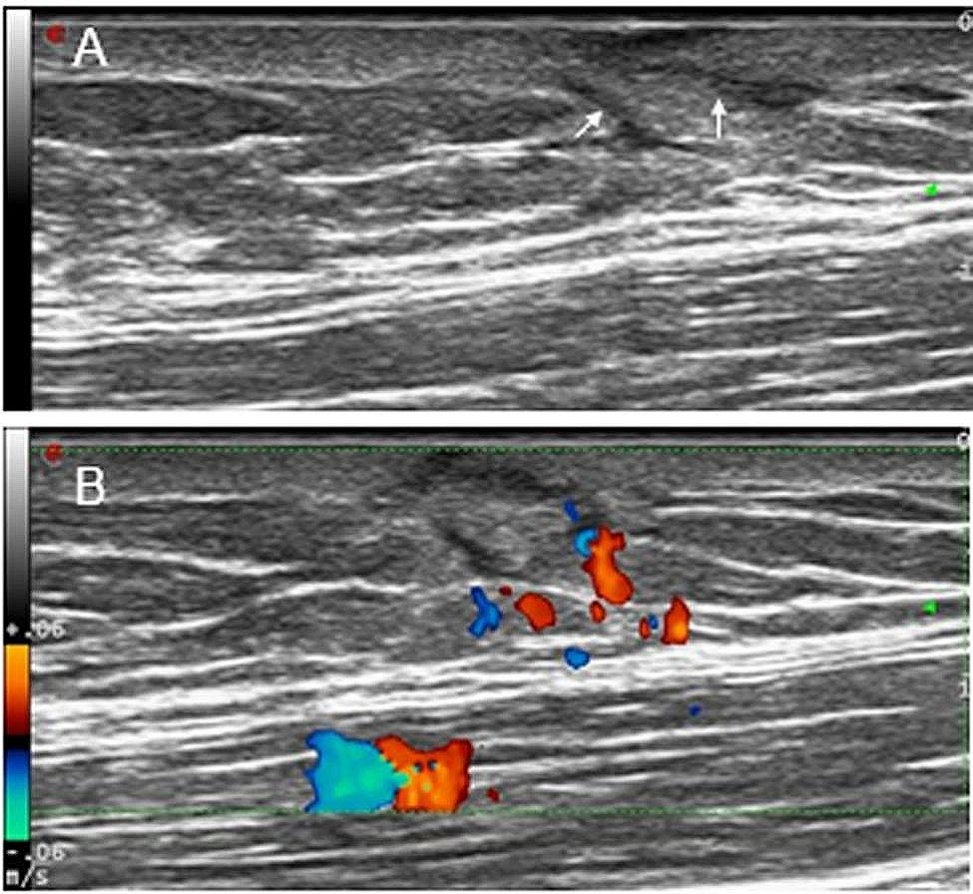
**A** A 32-year-old female with recurrent DFSP in the abdominal wall, transverse ultrasonogram revealing an ill-defined, hypoechoic, heterogeneous, and subcutaneous lesion, forming hypoechoic with finger-like projections (arrows). **B** Color Doppler ultrasonogram revealing rich blood flow signal

Gray-scale and color Doppler US characteristics are summarized in Table 2. For the maximum diameter, 22 recurrent DFSPs and 13 scars were measured as 31.2 ± 15.3 mm and 17.4 ± 11.5 mm, respectively. Twenty-five recurrent DFSPs and 30 postoperative scars underwent CEUS examination, and 9 postoperative scars showed no entry of contrast agent. CEUS features are detailed in Table 3. Representative sonographic findings for recurrent DFSP and postoperative scar are shown in Figs. 1, 2, 3, 4 and 5.

**Table 2:** Summary of gray-scale and color Doppler ultrasound characteristics of recurrent deep dermatofibrosarcoma protuberans (DFSP) and post-resection scar

Variables	Recurrent DFSP	Post-resection scar	*P*
Lesion No.	34	38	
Depth, mm	14.5 ± 10.9	6.0 ± 4.9	0.004
Shape			
Regular	12	25	0.010
Irregular	22	13	
Border			
Well-defined	14	34	0.000
Ill-defined	20	4	
Echogenicity			
Hyperechoic	2	0	0.425
Hypoechoic	22	35	0.004
Hypoechoic with finger-like projections	5	0	0.047
Mixed echoic	5	3	0.294
Echotexture			
Homogeneous	7	35	0.000
Heterogeneous	27	3	
Vascular density (/cm^2^)	2.79 ± 1.09	0.53 ± 0.60	0.000
Blood flow on CDFI			
No (Alder 0)	0	17	0.000
Not rich (Alder 1)	9	16	
Rich (Alder 2–3)	25	5	
Arterial flow	34	5	0.000
Venous flow	34	18	0.000

*CDFI* color Doppler flow image

**Table 3:** Contrast-enhanced ultrasound findings of recurrent dermatofibrosarcoma protuberans (DFSP) and post-resection scar

Indicator	Recurrent DFSP	Post-resection scar	*P*
Lesion No.	25	21	
Homogeneity, n (%)			
Homogeneous	7 (28)	21 (100)	0.000
Heterogeneous	18 (72)	0 (0)	
Enhancement intensity, n (%)			
Iso-enhancement	3 (12)	16 (76.2)	0.000
Hyper-enhancement	22 (88)	5 (23.8)	
Time Peak (s)	38.5 ± 15.7	44.1 ± 22.9	0.133
Peak	23.5 ± 9.4	15.1 ± 8.7	0.016
Sharpness	0.104 ± 0.032	0.025 ± 0.066	0.000

## Discussion

In this study, recurrent DFSP appeared on conventional US as a deep, irregular, heterogeneous, hypoechoic, ill-defined mass with finger-like projections, with rich arterial and venous flow; CEUS revealed heterogeneous hyper-enhancement, high peak and sharpness. Postoperative scars appeared on conventional US as shallow, hypoechoic, homogeneous lesions with well-defined borders and poor blood flow, and CEUS more commonly revealed homogeneous iso-enhancement, low peak and sharpness.

US is the first-line modality for evaluation of soft tissue lesions. One recent study [12] reported that the characteristic gray-scale US findings of DFSP are closely related to its pathological findings, such as marginal infiltration and tumor composition. Additionally, color Doppler techniques can increase the specificity of US by providing a real-time evaluation of vascularity [19], and the evaluation of nodular lesions of the skin [20, 21]. Therefore, US can be used as a routine examination for recurrent DFSP and postoperative scar. One recent study [13] reported the ultrasound findings of 35 recurrent DFSP cases, finding that recurrent DFSP lesions were commonly irregularly shaped, hypo-echo, and hyper-vascular on US images. Our findings were consistent with these. Hypoechoic with finger-like projections, a typical ultrasound manifestation of DFSP [22], was showed in 14.7% (5/34) of our recurrent cases, which was consistent with Zou et al. [13] reported and not very common in recurrent DFSP group.

On pathology [23, 24], DFSP showed high cellularity with slender spindle cells arranged in a distinct storiform pattern, which is consistent with its solid hypoechoic image. Furthermore, DFSP showed tumor cells infiltrating into the surrounding subcutaneous fat, which is consistent with its finger-like projections. Rich vascularization could support the aggressive growth patterns of DFSP, and the hypervascularity of DFSP is consistent with hyperplasia of small blood vessels on pathology [25, 26].

In our study, we found that vascularization could be an important feature distinguishing recurrent DFSP from scars, and CEUS could show microvascular circulation more clearly. The principle of CEUS is the introduction of contrast agent through different pathways to increase contrast within the tissues and improve the imaging of tissues, organs, and lesions [27]. Furthermore, the use of preoperative CEUS to improve the precision of DFSP resection was reported by Ma and in our previous study [14, 15]. The results of our study suggest that CEUS can provide valuable information distinguishing recurrent DFSP from post-resection scars; 30% of postoperative scars showed no entry of contrast agent, and recurrent DFSP was more likely to show heterogeneous and hyper-enhancement relative to normal peripheral soft tissue, which was possibly associated with the presence of necrosis or mucus components, and some vascularization, respectively, in the tumor. Along with increased postoperative time, scar tissue will be accompanied by connective tissue hyperplasia and reduced blood vessel numbers; however the blood supply of recurrent DFSP is relatively abundant. Recurrent DFSP presented a higher peak, which could be related to its higher blood vessel density than that of scars. The higher sharpness of recurrent DFSP could be related to its higher arterial blood flow velocity and rapid accumulation of lesional contrast agent, leading to early high levels of enhancement and a “fast rising” branch. Therefore, CEUS provided more evidence for the differential diagnosis of recurrent DFSP and postoperative scar.

We did not analyze a higher frequency (15 MHz or higher) probes evaluation in our study. In other series, however, high-resolution US is playing a growing role in the assessment of diagnosed melanoma cases and follow up [21]. Others have reported high-frequency transducers offer a remarkable detail of the skin abnormalities of the breast and axilla and superficial breast parenchyma abnormalities [28].

This study has certain limitations. First, although the presence of recurrent DFSP and postoperative scar could be suggested on US, it was difficult to evaluate its precise nature with larger lesions. Second, the present case series constitutes a single-center study with a small sample size; multi-center studies with larger sample sizes are needed for continued research. Third, we did not apply the new microcirculation software (superb vascular imaging, SMI and other). SMI examination is non-invasive and promising technique in the study of dermis abnormalities [29]. In future work, we will continue to collect more cases of DFSP by appropriate use of the SMI.

In conclusion, conventional and contrast-enhanced ultrasound produced distinct features of recurrent DFSP and post-resection scar, which could improve the accuracy of differential diagnosis.

## Data Availability

All data generated or analyzed during this study are included in this published article. Immunohistochemistry data of macrophage markers will be made available upon reasonable request to the corresponding author.

## Abbreviations

DFSP: Dermatofibrosarcoma protuberans
US: Ultrasound
CEUS: Contrast-enhanced ultrasound
CDFI: Color Doppler flow imaging
ANOVA: One-way analysis of variance
